# Targeting Androgen Receptor as a Novel Radiosensitizing Therapy to Improve Long-Term Survival and Anti-tumor Immunity in Glioblastoma via TGF-β/Smad3 Axis Reprogramming

**DOI:** 10.21203/rs.3.rs-8585892/v1

**Published:** 2026-02-05

**Authors:** Chi Zhang, Jyoti Kaushal, Nan Zhao, Rubayat Khan, Kan Liu, Chi Zhang, Fei Wang, Jie Chen, Bingjie Guan, Shuo Wang, Tom Hei, Tony Wang, Yuguo Lei, Michele Aizenberg, c lin

**Affiliations:** Mayo Clinic Arizona; University of Nebraska Medical Center; University of Nebraska Medical Center; University of Nebraska Medical Center; University of Nebraska - Lincoln; University of Nebraska - Lincoln; University of Nebraska Medical Center; University of Nebraska Medical Center; United Hospital of Fujian,; University of Nebraska Medical Center; Columbia University Medical Center; Columbia University Irving Medical Center; Pennsylvania State University, University Park; University of Nebraska Medical Center; university of Nebraska Medical Center

**Keywords:** glioblastoma, AR antagonists, radiotherapy, TGF-β/Smad pathway, LIF/STAT3 pathway, tumor microenvironment

## Abstract

Glioblastoma (GBM) remains the most aggressive primary adult brain cancer with limited therapeutic options. Our prior research demonstrated that androgen receptor antagonists (ARAs) enhance survival in GBM mouse models by preferentially suppressing glioma stem cells. This study investigates the potential of ARAs as radiosensitizers in combination with radiotherapy (RT). Combined effects of ARAs and RT were evaluated using an orthotopic GBM mouse model. RNA-Seq and TCGA analyses delineated AR-associated regulatory networks, while integrated cellular and molecular approaches utilizing human and mouse GBM cell lines, as well as in patient-derived primary high-grade glioma cultures, interrogated signaling and immune paradigms. ARA treatment induced G2/M cell cycle arrest, apoptosis, and downregulated DNA repair genes, with AR expression correlating with the DNA repair and TGF-β pathway. In both immortalized GBM cell lines and primary high-grade glioma cultured cells, ARAs in combination with RT showed only a modest enhancement of radiosensitivity. However, in an orthotopic GBM mouse model, ARA + RT demonstrated a strong synergy, achieving 100% long-term survival, compared to less than 50% with ARA alone and 0% with RT alone. Mechanistically, ARA modulated the TGF-β pathway, durably switching from Smad3 linker to c-terminal phosphorylation (pSmad3C) and inhibiting LIF/STAT3 axis. Distinct TGF-βs ligand expression patterns were observed in ARA-treated GBM cells. A protein–protein interaction noted between AR and Smad3, which was disrupted following ARA treatment, leading to elevated protein levels and nuclear localization of pSmad3C (S423/425), indicating normalization/activation of the TGF-β/pSmad3C-dependent anti-tumorigenic cascade. Comprehensive analyses in GBM cell lines and mouse model tissues demonstrated pathway reprogramming characterized by elevated TGF-β2 and increased pSmad3C. Tissue analysis revealed immune activation in the tumor microenvironment, while peripheral blood and spleen showed systemic immune responses following ARA + RT. Our study provides novel insights into how ARAs enhance RT efficacy through immunomodulation involving TGF-β/pSmad3C cascade, offering therapeutic implications in GBM.

## Introduction

Glioblastoma (GBM) is the most common and aggressive type of primary brain malignancy in adults. The standard of care for this tumor is maximal safe surgery followed by radiation therapy (RT) with concurrent and adjuvant temozolomide, an alkylating chemotherapy^1,2^. RT remains a key treatment for newly diagnosed and recurrent GBM. Despite advances such as modern RT techniques, temozolomide, and salvage therapies, prognosis remains poor, with a median survival of under two years and few long-term survivors. GBM recurrence is largely driven by therapy-resistant cancer stem cells and an immunosuppressive microenvironment. GBM is highly genomic and transcriptionally heterogeneous, yet targeted and immunotherapies have shown limited survival benefit in clinical trials^3–8^. Identifying novel targets to develop effective radio- or chemotherapy sensitizers that eliminate cancer stem cells or modulate the tumor microenvironment is crucial for improving clinical outcomes in GBM.

Our group, as well as others, have demonstrated that GBM tumors commonly overexpress AR in both genders and the remarkable tumor suppressive effects of ARAs, bicalutamide (BIC), and enzalutamide (ENZA), which work equally well in cultured GBM cells^9–11^. We have also shown previously in an orthotopic mouse model that ARAs, as monotherapy, significantly prolonged the survival of GBM-carrying mice^12^. Our studies have confirmed the preferentially suppressive effects of AR on glioma CSCs under both *in vitro* and *in vivo* conditions^12^. ARAs could potentially serve as a novel class of drugs with radiosensitizing effects against GBM, as indicated by the data from Werner *et al*. and Zalcman *et al*., although only in nude mouse models^9^. Our previous RNA-seq studies, coupled with Gene Set Enrichment Analyses (GSEA), showed that the transforming growth factor-beta (TGF-β) signaling pathway is among the top three pathways most significantly regulated in U87MG cells after ARA treatment. With the significance of TGF-β signaling, particularly the TGF-β/LIF-LIFR/STAT axis, contributing to not only CSC development but also immunosuppression in the GBM tumor microenvironment (TME)^13–19^, our group has further explored the AR antagonists as radiosensitizers in a syngeneic orthotopic GBM mouse model, focusing on TGF-β signaling and ARA-induced immunomodulation.

## Materials and Methods

### Cell culture and combenefit study of radiation and ARAs on GBM cells in vitro

GBM cell line details are listed in Table S1, and cell were cultured following a previously published protocol^12,20^. Primary high-grade glioma (HGG) was derived from freshly dissected tumor tissue from a male patient with a newly diagnosed astrocytoma, WHO grade 4, IDH1 mutant under approved protocol by Institutional Review Board (IRB # 0198–19-EP). Primary cultures were established following the lab protocol, employing high-glucose DMEM supplemented with Neurocult growth factors, heparin, bFGF, and EGF. Human cortical neuronal (HCN-2) cells were obtained from ATCC (Catalog #: CRL-10742) and cultured according to the recommended protocol. Normal Human Astrocytes (NHA/ ASTRO) were gifted from Dr. Sidharth Mahapatra at the University of Nebraska Medical Center, initially purchased from Lonza Bioscience (Walkersville, MD), and grown in ABM basal medium supplemented with growth factors (Lonza Biosciences)^22^. All cells were maintained in a 95% humidity environment at 37°C with 5% CO_2_.

Graded doses of radiation (2 Gy, 4 Gy, 8 Gy, and 16 Gy) were combined with different concentrations of ENZA (10 μM, 20 μM, 40 μM, and 80 μM) or BIC (40 μM, 80 μM, 120 μM, 180 μM, and 240 μM) to study potential synergism between radiation and AR antagonists using the Loewe model^20^. Cell viability was evaluated 3 days after radiation using the Cell Titer Blue Assay (Promega, Madison, WI, USA). Combenefit analyses were conducted using the Combenefit software developed by University of Cambridge^21^.

### 3-D culture/Single-spheroid culture

For spheroid formation, we followed the published protocol with modifications^12^. Human GBM cells and primary HGG cells were cultured in 96-well low-attachment U-bottom plates at a density of 200 or 500 cells per well to promote spheroid formation. Culture media were used according to the cell line requirements and treated with ARA as specified in the experiment group setup. U87MG cells were maintained in DMEM supplemented with 10% FBS, while primary cultures were grown in media containing specific supplements. Spheroid growth and diameter were monitored daily or on alternate days under a microscope. Growth curves were generated and compared between groups using ImageJ software for quantification of spheroid size.

### Colony Formation (clonogenic assay)

Cells were seeded in 6-well plates at densities ranging from 500 to 10,000 cells per well in triplicate, depending on each cell line’s growth characteristics and colony-forming capacity. ARAs were added at the time of seeding, and the culture medium was replenished once for GBM cell lines or twice for primary human HGG cells during the course of the experiment. Cells were incubated for 10–20 days at 37°C in a humidified atmosphere containing 5% CO_2_. At the end of the incubation period, colonies were fixed with ice-cold methanol and stained with a crystal violet solution (0.125 g crystal violet in 50 mL of 20% methanol).

### DNA Repair Gene Expression Analysis with The Cancer Genome Atlas (TCGA) Database

Spearman’s rank correlation coefficients of mRNA expression levels between AR and DNA repair genes based on RNA-seq results from TCGA database of GBM patients were calculated by GEPIA (http://gepia.cancer-pku.cn/)^23^.

### RNA-seq

U87MG cells were treated with 80 μM ENZA for 4 and 48 hours before the total RNA was isolated using RNeasy Plus Mini Kit (Qiagen, Netherlands). The RNA-seq was performed by next-generation sequencing (NGS) using NextSeq550 (Illumina, San Diego, CA). Data analyses were performed per our previous study protocols^12^.

### Syngeneic orthotopic GBM mouse model

MGPP-3 (PDGF+, P53 −/−, PTEN −/−) which is a murine GBM cell line was kindly provided by Dr. Peter Canoll (Columbia University, New York, NY, U.S.) and previously studied in our laboratory^12,24,25^. This cell line was generated from primary cultures from a mouse GBM tumor model induced by injection of VSVG-pseudotyped PDGF-IRES-Cre (PIC) retrovirus, which expresses PDGF and Cre in one transcript, into rostral subcortical white matter (WM) of transgenic mice that carry floxed Pten and p53 (Pten^f/f^; p53^f/f^), and stop-floxed luciferase reporter. 5 × 10^4^ MGPP3 murine GBM cells were implanted into the right hemisphere of 16–17-week-old male mice (20–30 g), and tumor growth was monitored weekly using In Vivo Imaging System (IVIS), as previously described^12^. Mice were regrouped and received intraperitoneally injection into the mice three times per week following the published protocols^26^. Details are provided in the Supplementary Methods.

### Magnetic Resonance Imaging (MRI)

Mice treated with vehicle, BIC, RT, or BIC + RT were imaged by MRI (5 weeks after treatment). Anesthesia was induced with 1.5% isoflurane in a 70% nitrous oxide/30% oxygen mixture, prior to MRI acquisition. Details are provided in the Supplementary Methods^27^.

### Quantification of bicalutamide concentrations in tissues with Ultra-Performance Liquid Chromatography (UPLC)

Plasma (100 μL) or brain tissue (50 mg homogenized in 500 μL water) was mixed with 1 mL acetonitrile containing Tolbutamide (20 μL, 15 μg/mL), vortexed, and centrifuged. The supernatant was evaporated to dryness, reconstituted with 100 μL mobile phase (50:50 acetonitrile: water), and analyzed by UPLC. Details are provided in the Supplementary Methods.

### Immunohistochemistry

Slides prepared from Formalin-Fixed Paraffin-Embedded (FFPE) mouse GBM tissues were stained with antibodies (Table S2) individually or in combination, following published protocols^12^. Stained slides were scanned at 400x using the Ventana iScan HT and quantified with Definiens Tissue Studio (Ventana, Munich, Germany). Tissue sections stained with dual stain (purple and brown) were analyzed and quantified using ImageJ software. Specific plugins were employed to separate and measure individual staining channels, allowing for precise quantification of staining intensity, percentage of positive cells.

### Western blotting

After treating cells, cell pellets were homogenized in RIPA Lysis and Extraction Buffer containing protease inhibitors, and protein isolation, quantification, and processing were performed as previously described^12^. Details of the antibodies used in this experiment are listed in Table S2.

### Immunoprecipitation

Protein samples were first pre-cleared by incubation with Protein A/G plus agarose beads for 1–2 hours, followed by washing and centrifugation. The pre-cleared lysates were then incubated overnight at 4°C with continuous agitation with either specific antibodies or isotype controls, along with 60 μL of Protein A/G plus agarose beads. The following day, the samples were centrifuged, and the pellets were washed twice and resuspended in RIPA lysis buffer. Immunoprecipitated proteins, along with input controls (2% of total protein) and IgG control samples prepared in Laemmli buffer, were subsequently separated by SDS-PAGE^28^.

### Flow cytometry of tumor-infiltrating immune cells from mouse tissues

Mice were sacrificed four weeks after implantation for flow cytometry analysis of tumor-infiltrating immune cells. Details are described in the Supplementary Methods and were conducted according to the protocols outlined^29,30^ and utilized antibodies listed in Table S3.

### Confocal microscopy

Cells were cultured on coverslips, fixed with 4% p-HCHO, permeabilized, and blocked with 5% BSA and processed per our previously published protocol^31–34^.^31–34^. Cells were mounted with DAPI FluoromountG (Southern Biotech). Confocal images were captured using a Zeiss LSM710 microscope.

### Computational analysis of immune infiltration in GBM TME

RNA-seq data from TCGA GBM patients were analyzed using TIMER2.0 (http://timer.cistrome.org/), which employs a tissue-specific algorithm to estimate immune cell infiltration^35^.

### Statistics

Apoptosis, cell cycle, and immunohistochemistry (IHC) data were calculated as Mean ± SEM. Statistical analyses used Student’s t-test (two groups) or one-way ANOVA (more than two groups) using GraphPad (Version 8.3.1, San Diego, CA), and Kaplan-Meier analysis for overall survival. Significance was set at p < 0.05.

## Results

### Enhanced but Limited Synergy of ARAs Combined with Radiotherapy on Human and Mouse GBM Cell Lines and Primary HGG Cultures

**Figure 1:**
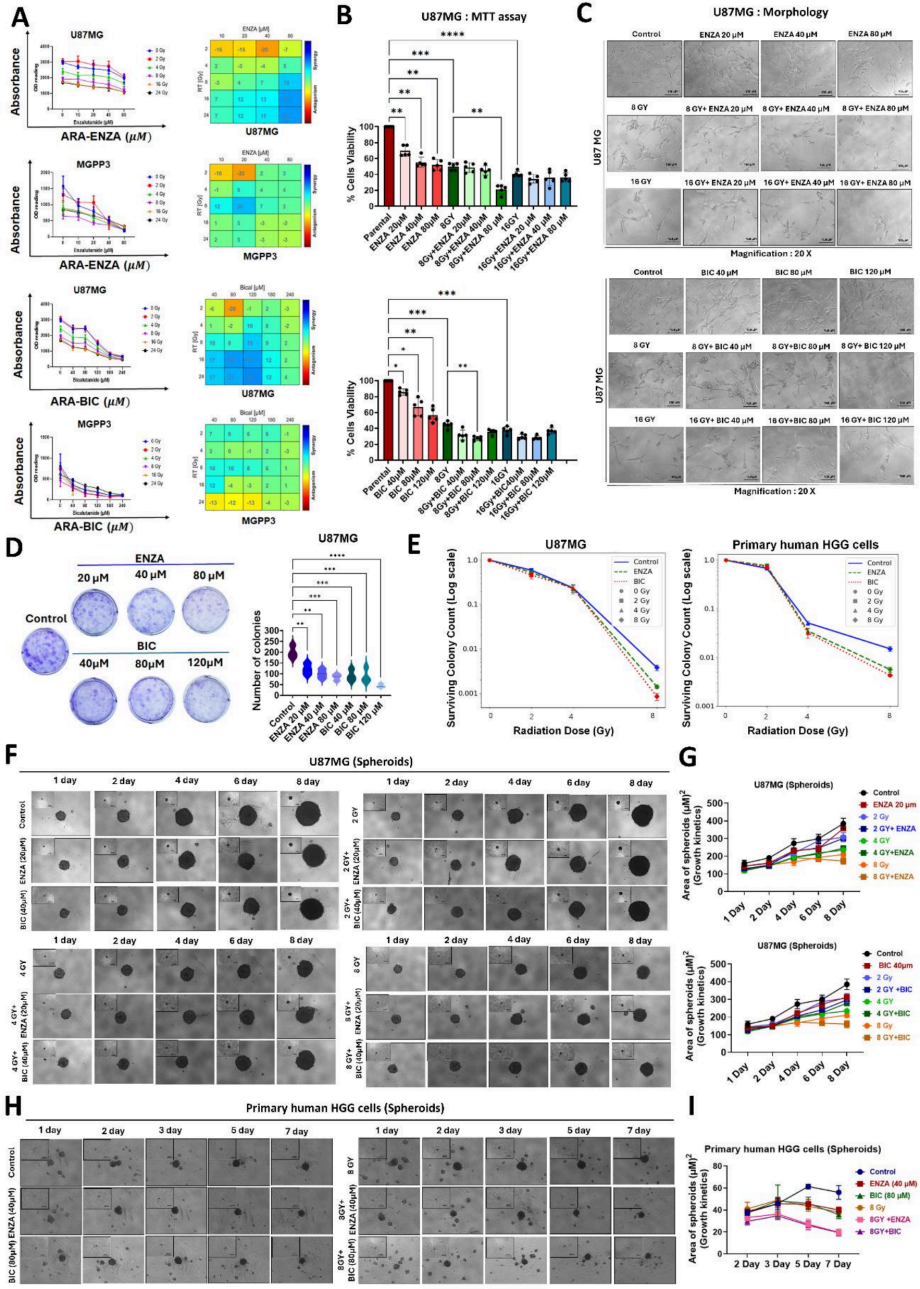
Synergistic effects of Enzalutamide or bicalutamide and radiation on human, mouse GBM cell line and primary HGG cells **(A)** Cell survival curve of U87MG and MGPP3 after the treatments of ENZA or BIC and radiation (left panel). Loewe synergy and antagonism matrix of ENZA or BIC and radiation at indicated concentrations and doses on the growth of U87MG and MGPP3 cells (right panel). The synergy scores shown can be interpreted as the average excess response due to treatment interactions, i.e., a synergy score of 23 corresponds to 23% of response beyond expectation. **(B)** Cell viability was assessed using the MTT assay in U87MG cells treated with various concentrations of ARAs (ENZA or BIC) and in combination with radiation doses (8 Gy or 16 Gy). Data are expressed as mean ± SEM (n = 5). Statistical significance was defined as P ≤ 0.05. For group comparisons, *P < 0.05, **<0.01, ***<0.001 for treated group vs. untreated control cells. **(C)** Representative images show morphological alterations and reduced cell growth across the different treatment groups as presented. **(D)** Evaluation of in vitro colony-forming ability in U87MG cells treated with different concentrations of ENZA (20 μM, 40 μM, and 80 μM) or BIC (40 μM, 80 μM, and 120 μM) (left panel). Quantitative analysis of colony numbers is shown (right panel), with data presented as mean ± SEM (n = 5). **(E)** Quantitative analysis of colony formation in human GBM cells (U87MG, left panel) and primary cultured HGG cells (right panel) treated with ARAs in combination with varying radiation doses (2 Gy, 4 Gy, and 8 Gy). **(F)** Representative images showing the day-dependent growth of U87MG spheroids treated with different doses of radiation (2 Gy, 4 Gy, and 8 Gy) in combination with ARAs (ENZA (20 μM); BIC (40 μM)) compared to treatment with ARAs alone, radiation alone, or the untreated control group. **(G)** Quantitative assessment of spheroid growth under different treatment conditions. Results are expressed as mean ± SEM (n = 3). **(H)** Representative images showing the day-dependent growth of primary human HGG spheroids treated with 8Gy doses in combination with ARAs (ENZA (40 μM); BIC (80 μM)) compared to treatment with ARAs alone, 8Gy alone, or the untreated control group. **(I)** Quantitative assessment of spheroid growth under different treatment conditions. Results are expressed as mean ± SEM (n = 3).

We have previously reported that ENZA can inhibit the proliferation of U87MG and MGPP3 cells in a dose-dependent manner^12^. To test the potential synergistic effect of ENZA, BIC, and RT on tumor cell killing, we performed the combenefit analysis using an online Combenefit software developed by the lab of Dr. DiVeroli Giovanni^21^. In the U87MG cell line, ENZA had the strongest but a mild synergistic effect at a concentration of 80 μM when combined with radiation at a dose ranging from 8Gy to 24 Gy. The synergy scores ranged from 18 to 23, *i.e*., only 18 to 23% of extra inhibition of cell proliferation beyond expectation^36^ (Fig. 1A). A noticeable synergistic effect was observed in the MGPP3 cell line when 20 μM ENZA was combined with 8 Gy of radiation, with a synergy score of 20, again, indicating mild synergy between ENZA and radiation (Fig. 1A). For BIC, the most significant synergistic effects were observed when 80–120 μM BIC was combined with 8–24 Gy of radiation in the U87MG cell line (Fig. 1A). However, in MGPP3 cells, no obvious synergistic effect of BIC combined with radiation was observed with only likely some additive effects seen when the drug was combined with 8–12 Gy radiation with synergy scores between − 4 to 10 (Fig. 1A). These effects were further validated using an additional cytotoxicity assay (MTT and CTB), which demonstrated a significant additive effect in suppressing cell growth following 8 Gy radiation combined with ARAs treatment (Fig. 1B; Fig. S1A), accompanied by distinct morphological alterations indicative of growth suppression/cell death (Fig. 1C). Additionally, ARAs + RT induced growth suppression and morphological changes in primary human HGG cells (Fig. S1B). Additionally, we observed no cytotoxicity in human cortical neuronal (HCN-2) and astrocyte (ASTRO) cells at concentrations up to 200 μM (Fig. S1C).

Next, treatment with ARAs, including ENZA and BIC, resulted in a dose-dependent decrease in the clonogenic potential of human GBM cells (Fig. 1D). The radiosensitizing effect was further confirmed by colony formation assays, which demonstrated significantly reduced colony formation following combined treatment with 8 Gy + ENZA (20 μM) or 8 Gy + BIC (40 μM) in U87MG cells (Fig. 1E left panel, Fig. S1D), and 8 Gy + ENZA (40 μM) or 8 Gy + BIC (80 μM) in primary human HGG cells (Fig. 1E right panel, Fig. S1E). Furthermore, using a 3D single-spheroid culture platform for U87MG (Fig. 1F–G) and primary human HGG cells (Fig. 1H–I and Fig. S1F), we observed a significant sensitizing effect when 8 Gy RT was combined with ARAs. Notably, in both the clonogenic and spheroid assays, radiosensitization was more pronounced in primary HGG cells compared to U87MG cells. Altogether, based on multiple assays in both 2D and 3D culture systems, as well as across a range of human and mouse cell lines and primary cultures, we conclude that ARAs exert a radiosensitizing effect that ranges from mild to moderate.

### Synergistic Induction of Apoptosis by Combined RT and ARA Treatment with ARA enriches Cell Cycle to G2/M

**Figure 2:**
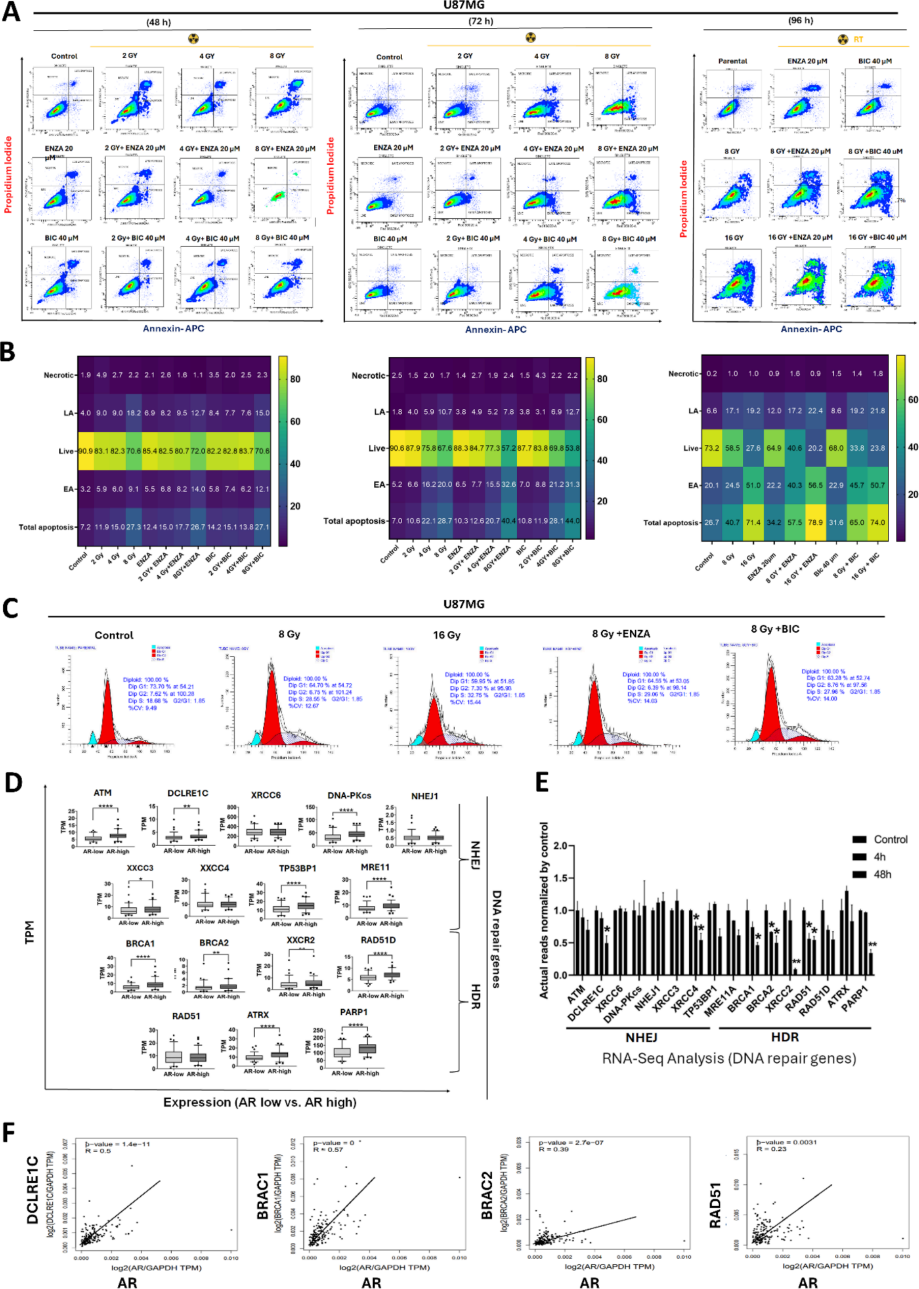
ARAs potentiate radiation-induced cytotoxicity via stimulation of apoptosis, cell cycle regulation, and associated with DNA repair response **(A)** Representative flow cytometry plots showing apoptosis in U87MG cells following treatment with ENZA (20 μM); or BIC (40 μM) either alone, with radiation alone, or in combination with varying radiation doses at 48 h, 72 h, and 96 h, as indicated **(B)** Heatmap depicting the percentage proportion of cells in nacrotic, live, early apoptosis, late apoptosis, and total apotosis populations under the respective treatment conditions. **(C)** Representative flow cytometry profiles showing cell cycle distribution in U87MG cells following treatment with 8 Gy or 16 Gy irradiation, either alone or in combination with ARAs (ENZA (20 μM); or BIC (40 μM). **(D)** The positive correlations of mRNA expressions between DNA repair genes and AR from RNA-seq results in GBM patients from TCGA database. **(E)** mRNA expression levels of DNA repair genes in U87MG cells cultured *in vitro* before and after the treatment of ENZA (80 μM) from RNA-seq results from our laboratory. *p<0.05. **p<0.01;***p<0.001;****p<0.0001 **(F)** Correlation analysis of DNA repair genes and AR in GBM patients from TCGA database.

To further explore the potential mechanism underlying the radiosensitizing effect of ARAs, we performed apoptosis and cell cycle analyses. Initially, GBM cell lines (U87MG, U138MG, and Ln229) were treated with either DMSO or 80 μM ENZA for 1–4 days to assess apoptosis (Fig. S2A). In U87MG cells, over 90% of cells underwent apoptosis after 4 days of ENZA treatment. Ln229 cells exhibited a time-dependent increase in both early and late apoptosis following ENZA exposure. In contrast, U138MG cells showed significantly elevated apoptosis, but their levels did not increase with culturing time. These findings suggest ARAs have varying effects on GBM cell lines, warranting further investigation into the underlying mechanisms. GBM cells were subsequently treated with varying doses of radiation (2, 4, 8, and 16 Gy) in combination with ENZA (20 μM) or BIC (40 μM) for different time points (48, 72, and 96 h), and apoptosis was analyzed (Fig. 2A–B). Synergistic effects became apparent at 72 h with the 8 Gy combination, and after 96 h of incubation, total apoptosis increased to approximately 17% with ENZA and 25% with BIC, predominantly reflecting early apoptosis.

Next, cell cycle assays were performed with these GBM cell lines treated with ENZA (80 μM) for 48 hours, followed by flow cytometry analyses. All three cell lines showed cell cycle enrichment at the G2/M phase (Fig. S2B). After ENZA treatment for 2 days, G2/M phase distribution of cells significantly increased from 14% to 23%, 18% to 25%, and 9% to 15% in U87MG, U138MG, and Ln229 cell lines, respectively (Fig. S2B). Thus, ENZA treatment alone induced G2/M arrest. As expected, RT alone with either 8 Gy or 16 Gy led to S-phase cell accumulation (Fig. 2C). Combining ARAs with RT in cell cycle analyses revealed the dominant effects of RT, with significantly increased cell cycle arrest at the S-phase (Fig. 2C).

### Positive Correlation Between mRNA Expression of DNA Repair Genes and AR in GBM

To further elucidate the ARAs-mediated mechanisms underlying cell death, we also extended our investigation to explore DNA repair pathways and the role of AR in GBM. To explore the correlation between the expression levels of AR and DNA repair genes in TCGA database, we selected 16 wellknown DNA repair genes^37^. We found that the mRNA expression levels of 12 out of the 16 DNA repair genes were significantly higher in patients’ specimens with higher AR expression (AR-high) compared with those with lower AR expression (AR-low) (Fig. 2D).

Additionally, our RNA-seq of U87MG cells showed significant downregulation of DNA repair genes after 48h ENZA (80 μM) treatment (Fig. 2E). Strong correlations with AR were observed for DCLRE1C, BRCA1/2, and RAD51 at RNA expression levels (Fig. 2F).

### ARAs Significantly Sensitize Brain Irradiation and Extend Long-Term Survival in a GBM Mouse Model

**Figure 3:**
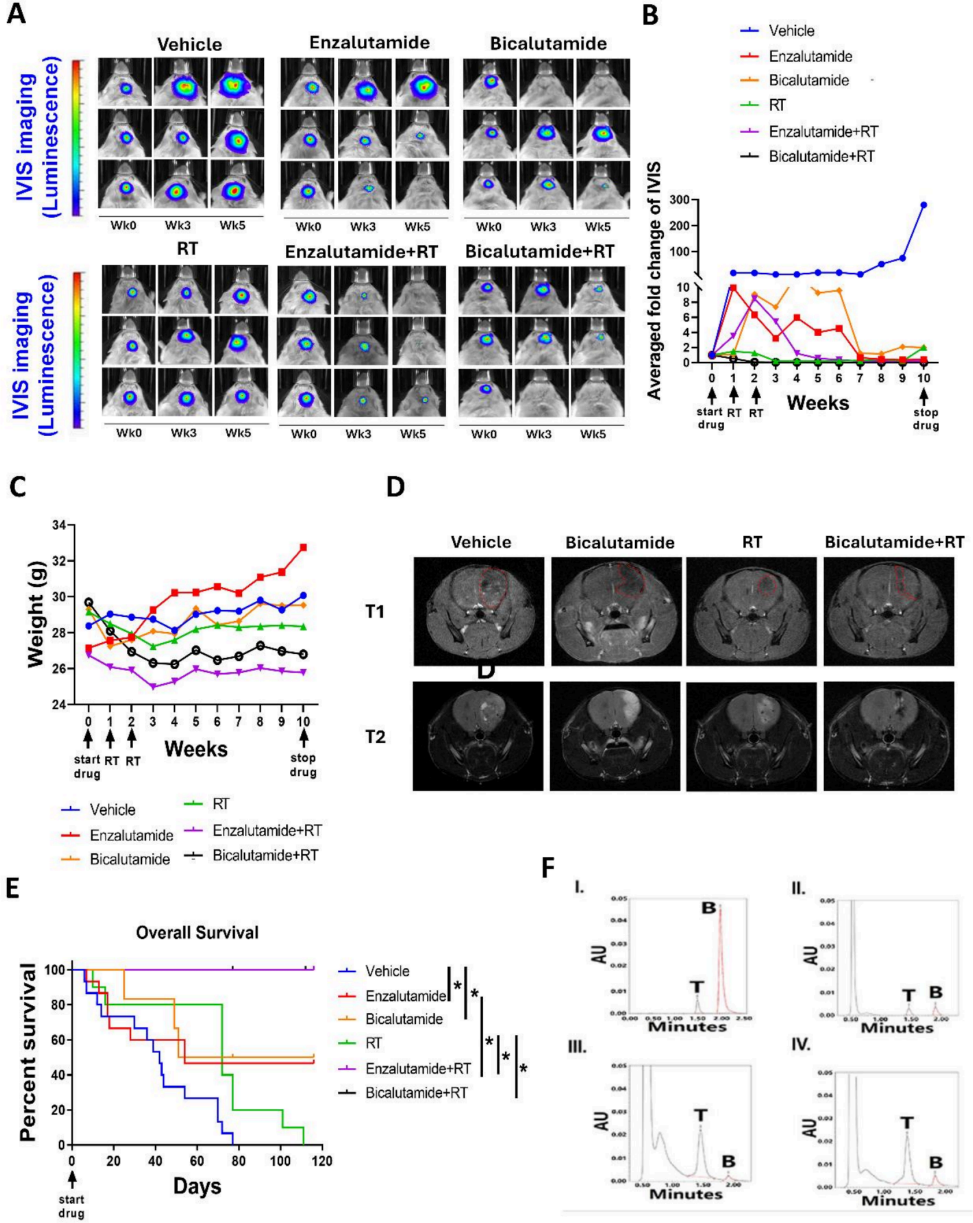
AR antagonists suppressed GBM tumor growth *in vivo* and prolonged overall survival in treated mice with MGPP3 cells implanted in brain. **(A)**Representative IVIS images of the tumor growth in the mouse brain. Week 0, 3, and 5: IVIS imaging taken prior to (week 0), 3, and 5 weeks after initiating drug injection. **(B)** Fold changes of the bioluminescent signals of the tumors in the brain after the treatment of vehicle only, ENZA (20 mg/kg, 3 times per week, IP), BIC (60 mg/kg, 3 times per week), RT only (10 Gy/fraction × 2 fractions), ENZA plus RT, or BIC plus RT. **(C)** Weight changes of the mice after the treatment. **(D)** Representative MRI images of the tumor growth as delineated by the red dotted lines in the mouse brain at week 11 after the treatment. **(E)** Overall survival was significantly improved in mice treated with ENZA/BIC with or without RT compared with RT only or vehicle only, respectively. *: p<0.05. **(F)** UPLC of BIC concentrations in the brain and plasma of male mice after treatment with 60 mg/kg BIC IP (equivalent to 300 mg for human) for 7 weeks (3 times per week). I. BIC (50 μg/ml) and tolbutamide (100 μg/ml) curves in the UPLC. II. plasma collected from a mouse at 24 h after the final treatment. III. normal brain collected from a mouse at 24 h after the final treatment. IV. tumor brain collected from a mouse at 24 h after the final treatment. T represents tolbutamide as internal standard; B represents bicalutamide.

We evaluated the combined effects of ARAs and RT in a syngeneic orthotopic GBM model by intracranially injecting luciferase-expressing MGPP3 cells. Starting five weeks post-inoculation, mice received ENZA (20 mg/kg), BIC (60 mg/kg), or vehicle three times weekly for 10 weeks. GBM tumor-bearing mice received whole brain RT at 10 Gy per fraction, once weekly for two weeks, based on *in vitro* data showing optimal ARA + RT synergy at about dose, and the reason that it equals the biological effective dose (BED) of the commonly practised whole brain RT dose in clinic (30Gy in 10 fractions). ARA or vehicle was administered *via* IP injection starting one week before RT. Tumor progression was monitored by bioluminescence imaging (Fig. 3A). Representative IVIS images and quantification for each treatment group are shown in Fig. 3A–B. The weight changes of the mice after the treatment in different groups were shown in Fig. 3C. Brain MRI revealed tumor shrinkage with BIC, or RT alone compared to vehicle, while BIC + RT led to complete tumor disappearance in all mice after 10 weeks (Fig. 3D). We found that GBM tumor growth was suppressed (size reduced or stabilized) at week 7 weeks after initiation of the drug in 5 out of 9 mice (55.6%) in the ENZA-treated group, 3 out of 6 mice (50%) in the BIC-treated group, 0 out of 9 mice (0%) in the vehicle only control group, 4 out of 10 mice (40%) in the RT only group, 7 out of 7 (100%) in the ENZA plus RT group, and 6 out of 6 (100%) in the BIC plus RT group. Mice treated with ENZA, BIC, RT, or their combinations showed significantly improved survival *versus* controls (p < 0.05). Median survival was 42 days (control), 54 (ENZA), 84 (BIC), and 101 days (RT). The median survivals were not reached in the ENZA plus RT and BIC plus RT groups with 100% OS after long-term follow-up (Fig. 3E).

### Quantification of Drug Concentrations in vivo

Bicalutamide concentrations were measured in brain tumor, contralateral normal brain, and plasma tissues after seven weeks of treatment using UPLC (Fig. 3F). Tumor tissue contained 5.21 μg/mg, approximately twice the concentration in normal brain tissue (2.55 μg/mg), and similar to plasma levels (5.12 μg/ml). Higher concentrations were found in extracranial tissues like the pancreas and liver (Table S4).

### Development of Long-Term Immunological Memory Against Tumors in Mice Surviving ARA or ARA + RT Treatment

To assess long-term immunity, surviving mice (8 months post-ARA or ARA + RT treatment) were rechallenged with MGPP3 cells in the contralateral brain hemisphere. After one month, tumor growth occurred in 1/4 BIC-treated mice, 0/6 BIC + RT mice, and 5/5 control mice, indicating durable antitumor immunity with ARA + RT (Fig. S3).

### ARA Treatment Modulates Smad3 Phosphorylation, Inhibits Phosphorylation of Signal Transducer and Activator of Transcription 3 (Stat3) and Switches TGF-β Signaling

**Figure 4:**
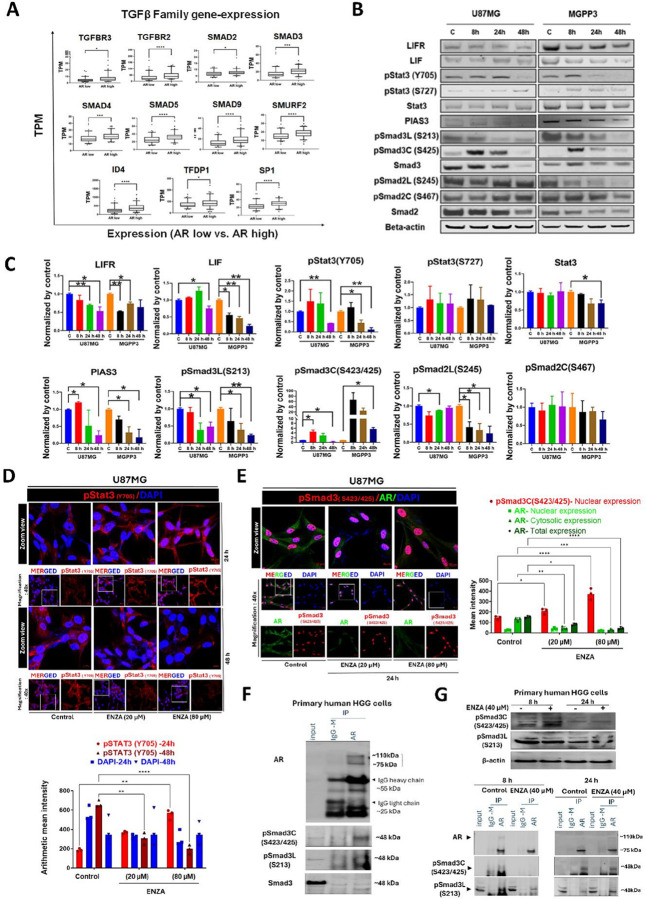
ARA treatment modulates the TGF-β/Smad3 and LIF/STAT3 cascades by inducing Smad3 and Stat3 phosphorylation and disrupting the physical interaction of pSmad3 with AR. **(A)** The mRNA expression level of AR positively correlates with the levels of genes involved in the TGF-β pathway in the TCGA database. *: p<0.05; **: p<0.01; ***: p<0.001; ****: p<0.0001. **(B)** Western blotting assays on selected proteins responding to ARA treatment in U87MG and MGPP3 cell lines. Protein level changes of genes from both LIF-STAT3 (top) and TGF-β (bottom) pathways before and after the treatment for 0 (control), 8, 24, and 48 hours. **(C)** Quantifications of the protein levels of genes in the LIF-STAT3 and TGF-β pathways before and after the treatment of ENZA. **(D)** Representative confocal laser scanning microscopy images display the expression patterns of pStat3 (Y705) in U87MG cells following treatment with 20 μM or 80 μM ENZA for 24 and 48 h (upper panel). Quantitative fluorescence intensity analysis of these images is presented (lower panel) for the specified groups (n=3). **(E)** Representative confocal images depict the subcellular distribution of pSmad3C (S423/425) and AR in U87MG cells treated with 20 μM or 80 μM ENZA for 24 h (left panel). Quantitative fluorescence intensity analysis of the confocal images is presented (right panel) for the specified groups (n=3). The data include nuclear expression of pSmad3C (S423/425) and cytosolic, nuclear, and total expression of AR at 24 h., based on mean intensity following ARA. **(F)** Representative WB image of AR, pSmad3C (S423/425), pSmad3L (S213), and Smad3 after AR enrichment via IP in primary human HGG cells. IgG-M denotes mouse IgG control. **(G)** Representative WB image of pSmad3C (S423/425), and pSmad3L (S213) in primary human HGG cells (whole cell-lysate) treated with ENZA (40 μM) and untreated control in a time-dependent manner (8 h and 24 h). β-Actin was used as a loading control (upper panel). Representative WB image of AR, pSmad3C (S423/425), and pSmad3L (S213) after AR enrichment via IP in primary human HGG cells- treated with ENZA (40 μM) at 8 h and 24 h compared to untreated control (lower panel).

All TGF-β pathway-associated and regulatory molecules are analyzed in TCGA GBM patients with respect to high *versus* low AR expression levels, as shown in Fig. S4. Furthermore, the significantly correlated genes with AR are illustrated in Fig. 4A for clarity. We found that patients with high AR expression levels had significantly higher expressions of the genes in the TGF-β pathway, except for the inhibitory Smads, *i.e*., Smad6 and 7 (Fig. 4A). However, among the three TGF-β ligands (TGF-β1, -β2, and -β3) and receptors (TGFβR1, R2, and R3), only TGF-β2 and TGFβR3 showed correlation in their expression levels to AR, with higher AR-expressing GBM demonstrating higher levels of TGF-β2 and TGFβR3 (Fig. 4A). The others are not significantly correlated to AR expression (Fig. S4). Compared with normal brain tissues, GBM tumor tissues also had significantly higher expression levels of AR, TGF-β genes, Smad2, and Smad4 but decreased expression of the inhibitory Smad 6 and Smad7 numerically (Fig. S5). Furthermore, we showed that the transcription levels of most of the genes decreased after AR antagonist treatment, including TGF-β receptors (TGFβR1, 2, and 3) and SMADs, in our previous publication^12^. However, TGF-β ligands, including TGF-β1, 2, and 3, were not significantly changed at their mRNA expression levels after ARA treatment, with TGF-β1 being the dominant expressed form in tumor cells. For functional validation, western blotting (WB) assays were performed with total proteins extracted from GBM cells treated with ENZA at a series of time points, focusing on TGF-β signaling/LIF and its receptor (LIFR)/STAT3 axis (Fig. 4B). ENZA treatment for 48 hours reduced LIF and LIFR expression in U87MG and MGPP3 cells. ARAs decreased Stat3 phosphorylation at Tyr705 but not Ser727, without affecting total Stat3 levels in U87MG cells. In MGPP3 cells, ARA also reduced Tyr705 phosphorylation, and total Stat3 protein levels were downregulated in a time-dependent manner after ENZA treatment (Fig. 4B). Protein inhibitor of Activated STAT3 (PIAS3) protein was also downregulated in a time-dependent manner after ENZA treatment for both cell lines. Levels of phosphorylation at the linker domain of Smad2 and Smad3 (pSmad2L (Ser245/250/255), pSmad3L (Ser213) decreased significantly after ENZA treatment in both MGPP3 and U87MG cells. Although the total protein of Smad2 decreased to some extent after ARA treatment in both cell lines, the pSmad2C (Ser467) was not significantly changed in either cell line after treatment. In contrast, the MH2 phosphorylation of Smad3 (Ser423/425) or pSmad3C (S423/425) at the c-terminus was increased notably in both U87MG and MGPP3 cells as early as 8 hours after ARA treatment, nearly simultaneously or slightly preceding pSmad3L downregulation but significantly earlier than pStat3 (Tyr705) (24–48 hours after ARA treatment). The quantitative analysis of these signaling molecules is represented in Fig. 4C. In addition, among the Smad family genes, Smad3 and Smad6 demonstrated the most drastic downregulation by ARA, particularly at 48 hours post-treatment, and Smad7 and 9 showed transient upregulation at mRNA levels (Fig. S6A left panel). Most of the Stat genes showed no or mildly decreased mRNA expression except for Stat4 and Stat5a which are both expressed at very low levels in U87MG cells, and Stat5b which is expressed at relatively high level but with drastic decreases in its expression 48 hours after drug treatment (Fig. S6A right panel).

Next, we performed confocal microscopy to examine the subcellular expression and colocalization patterns of these signaling components in the presence of ARA. Interestingly, we observed a transient increase in pStat3 (Y705) expression at 24 hours in U87MG cells treated with ARA, followed by a significant downregulation of phosphorylation levels at the extended time point of 48 hours, either at 20 μM or 80 μM (Fig. 4D). This pattern of pStat3 (Y705) response to ARA is very similar to LIF but delayed from pSmad3C changes based on WB results in U87MG cells (Fig. 4B), which is consistent with previously studied TGF-β/LIF/STAT3 signaling axis in GBM^16^. Confocal microscopy also demonstrated that ARA treatment for 24 hours promoted nuclear accumulation of pSmad3C (S423/425) while reducing cytosolic AR expression. Notably, higher ARA concentrations led to a predominant nuclear localization of pSmad3C (S423/425) and a marked decrease in AR expression (Fig. 4E). Additionally, we observed no alterations in total Smad3 expression after AR treatment for 24 hours with confocal microscopy (Fig. S6B). These fluorescence intensity-based expression findings are consistent with our WB results (Fig. 4B). Remarkably, immunoprecipitation analysis in primary human HGG cells revealed a predominant interaction between AR and the phosphorylated Smad3 (pSmad3C and/or pSmad3L), and no detectable binding to unphosphorylated Smad3 (Smad3) (Fig. 4F). Notably, analysis of whole-cell lysates showed increased pSmad3C but not pSmad3L levels following 8 h ENZA exposure compared to untreated cells, with the effects diminishing in 24 h-treated cells (top panel, Fig. 4G). Concurrently, 8 h ENZA treatment completely disrupted this interaction with pSmadC and AR but less prominent at 24 h (bottom panel, Fig. 4G). The interaction between AR and pSmadL form of Smad protein decreased significantly after 8 h ENZA treatment but was not completely abolished and was restored at 24 h. These results suggest that ENZA dissociates AR from binding to the MH2 domain at the c-terminus of Smad3, which is probably mostly unphosphorylated in tumor cells due to AR-binding/blockade, and it is the dissociation that facilitates Smad3C phosphorylation, potentially activating the pSmad3C–mediated signaling cascade.

### ARA Treatment Alone or in Combination with RT Enhances TGF-β2 Expression

**Figure 5:**
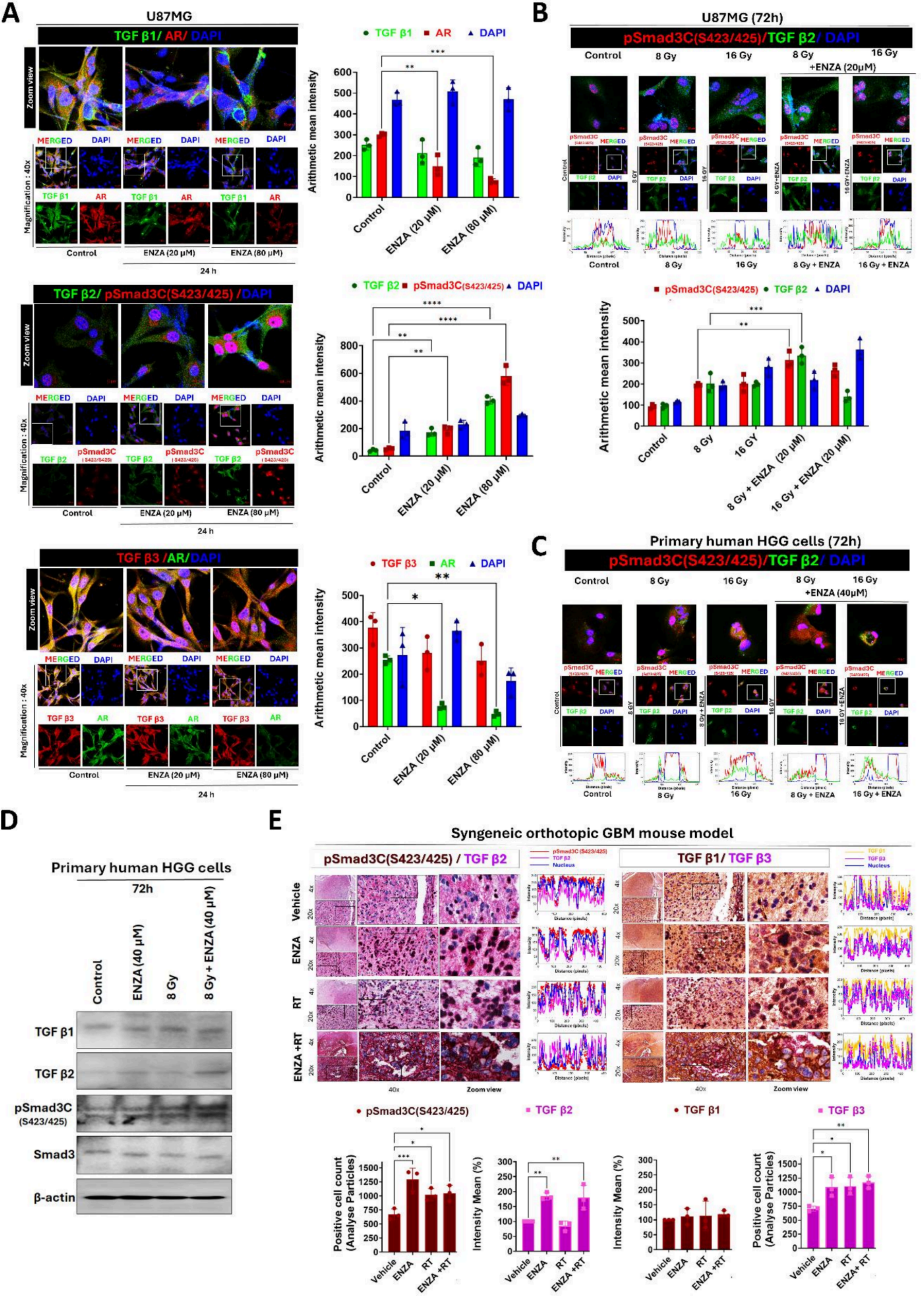
Impact of ARAs, alone or in combination with radiation, on the subcellular distribution of TGF-β ligands and pSmad3C in cell lines and mouse tissue models. **(A)** Representative confocal laser scanning microscopy images showing the expression levels and subcellular localization patterns of TGF-β1/AR; TGF-β2/pSmad3C (S423/425); and TGF-β3/AR in U87MG cells treated with 20 μM or 80 μM ENZA for 24 h (left panel). The quantitative fluorescence intensities of the confocal images are shown for the designated groups, as in (A) (n = 3) (right panel). **(B)** Representative confocal images showing the expression and subcellular localization of TGF-β2 and p-Smad3C(S423/425) in U87MG cells treated with ENZA (20 μM) alone, radiation alone (8 Gy or 16 Gy), or their combination. Cytosolic versus nuclear protein distribution was presented as a histogram at the single-cell level using intensity-distance analysis in ImageJ with the appropriate plugins. Additionally, the quantification of the mean intensity of overall expression was analyzed using ImageJ software (lower panel). **(C)** Representative confocal images showing TGF-β2 and pSmad3C (S423/425) localization in primary human HGG cells treated with ENZA (40 μM), radiation (8 or 16 Gy), or their combination. Cytosolic and nuclear distribution (intensity/distance in a single cell) was presented in a histogram using ImageJ. **(D)** Representative WB showing expression of TGF-β1, TGF-β2, pSmad3C (S423/425), and Smad3 in primary human HGG cells treated with ENZA (40 μM) alone, 8 Gy alone, their combination, or untreated control for 72 h. β-Actin was used as a loading control (upper panel). **(E)** Representative IHC images (upper panel) showing pSmad3C/TGF-β2 and TGF-β1/TGF-β3 in a syngeneic orthotopic GBM mouse model across different groups (vehicle, ENZA, RT, and ENZA+RT). Protein expression distribution was quantified in selected areas as histograms using intensity-distance analysis in ImageJ (plugins). Positive cell counts for pSmad3C (S423/425) and TGF-β3 were quantified based on analyzed particles, whereas TGF-β1 and TGF-β2 were quantified as mean percentage intensity relative to control (vehicle) in 40× images (lower panel).

We examined the TGF-β family members (TGF-β1, -β2, and -β3), along with the downstream signaling molecules, including Smad3, and their phosphorylation status by confocal microscopy. We found that pSmad3C was primarily localized in the nucleus (Fig. 5A); Smad3 was distributed in both the cytosol and nucleus (Fig. S6B); AR was detected in the cytosol and nucleus; and TGF-β family proteins were expressed in the cytosol (Fig. 5A). Notably, ENZA treatment (20 μM or 80μM for 24 h) resulted in a correlated and dramatic increase in pSmad3C (S423/425) and TGF-β2 expression, with a more pronounced effect observed in cells treated with 80μM. We did not observe significant changes in the TGF-β1 or β3 expression. Additionally, AR expression decreased in a dose-dependent manner with ENZA treatment (Fig. 5A). These results were validated *via* fluorescence quantitative analysis (Fig. 5A, right panel). The combination of RT + ENZA in U87MG and primary cultured HGG cells also resulted in increased expression of TGF-β2 and pSmad3C, with a more pronounced effect compared to single treatments (ENZA or RT alone) or untreated cells (Fig. 5B, C). This effect was evaluated across different radiation doses (Fig. S6C), with the most significant response observed at 8 Gy + ENZA. Results were similar between U87MG and primary HGG cells, with primary HGG cells showing even a stronger response compared to U87MG cells (Fig. 5B–C). Supportively, WB analysis revealed the most increased levels of TGF-β2 and pSmad3C in primary human HGG cells treated with 8 Gy + ENZA, while TGF-β1 and TGF-β3 levels remained unchanged (Fig. 5D).

Intriguingly, analysis of brain tissue from the orthotopic mouse GBM model (Vehicle, ENZA, RT, and ENZA + RT) with IHC revealed increased cytosolic TGF-β2 in the ENZA and ENZA + RT groups, along with nuclear accumulation of pSmad3C levels across all treated groups compared to the vehicle (Fig. 5E). TGF-β1 expression remained unchanged. These results were consistent with confocal microscopic and WB studies. However, IHC results on mouse brain tumor tissues demonstrated mildly but significantly elevated TGF-β3 expression after weeks of ENZA treatment *in vivo* (Fig. 5E), which was not observed with days of ENZA treatment *in vitro* in cultured cells (Fig. 5A).

### Correlation Between TGF-β Family Proteins, AR and Immune Infiltration

**Figure 6:**
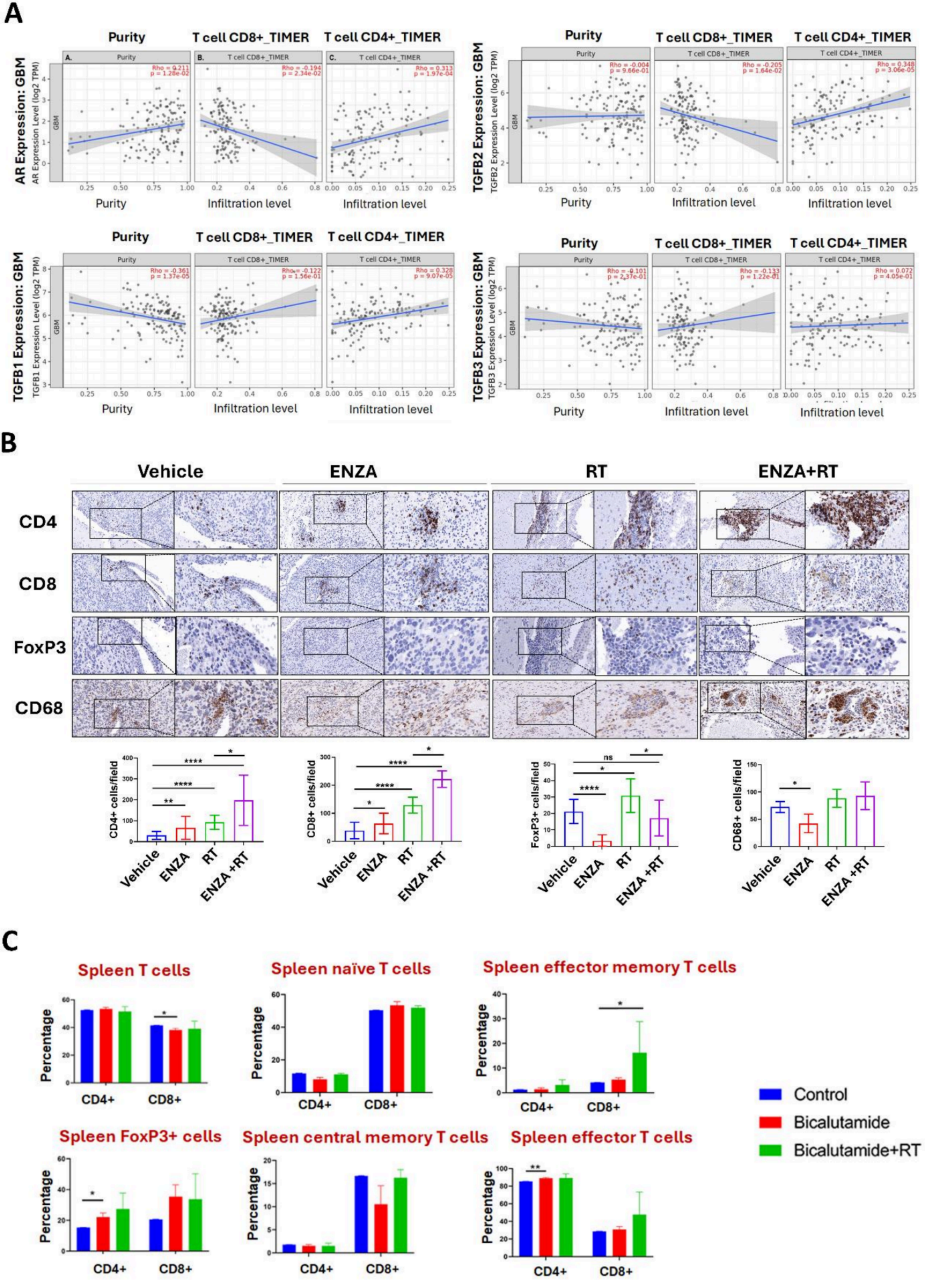
AR links to immune cell infiltration, and AR antagonist treatment remodels the tumor microenvironment by enhancing T-cell infiltration and potentiating radiation effects in brain tumors *in vivo*. **(A)** TIMER studies on AR/TGF-β1, 2, and 3 expression levels in GBM patients’ tumor specimens in the TCGA database. The correlations between the expression levels of AR, TGF-β1, -β2, and -β3 and tumor purity, CD8+ T cell infiltration, and CD4+ T cell infiltration were shown using Spearman’s correlation coefficient (Rho) and p values as labeled. **(B)**Representative images of IHC staining of the mouse brain tissue after ENZA treatment for CD4, CD8, FoxP3, and CD68 markers. For CD4, CD8, and CD68 markers, the left panels are shown at 200x magnification, with the boxed areas enlarged in the right panels (400x). Panels for FoxP3 marker staining on the left are 300x in magnification and 600x (upper panel). Quantifications of CD4+, CD8+, and regulatory T cells, and CD68+ tumor-associated macrophage cell densities in the brain tumors with or without treatments based on IHC staining results from A. *: p<0.05; **: p<0.01;****: p<0.0001 (lower panel). **(C)** Flow cytometry of immune cells in the spleen from the rechallenged mice with or without pretreated BIC ± RT. Percentage of CD4+ and CD8+ T cells, percentage of CD4+ and CD8+ T naive cells, and percentage of CD4+ and CD8+ T effector memory cells are shown (upper panel) and percentage of CD4+ and CD8+ regulatory T cells (Treg), percentage of CD4+ and CD8+ T central memory cells and percentage of CD4+ and CD8+ T effector cells are shown (lower panel). Analysis was performed in the peripheral blood of the mice 4 weeks after tumor rechallenging. *P < 0.05.

With observation of increased protein accumulation in the cytosol of TGF-β2, as the only TGF-β ligand among all three, after ARA treatment in cell cultures and the only one that correlates with AR expression level based on TCGA data, we further estimated immune cell infiltration based on AR and TGF-β, particularly TGF-β2 expression levels from RNA-seq results in GBM patients in the TCGA database (Fig. 6A)^35^. The computational algorithms, TIMER 2.0, were used to study AR expression level and its correlations with tumor purity and immune cell infiltration levels in GBM^38^. In GBM patients’ specimen, AR positively correlated with tumor purity, *i.e*., proportion of cancer cells in a sample (Spearman’s correlation coefficient (*Rho*): *R* = 0.211, p value: 0.0128). This indicates that AR expressions are less prominent in environmental cells including infiltrated immune cells than tumor cells. This is consistent with single-cell sequencing data from mouse brain tumors from our own studies showing that AR expresses in infiltrated T cells but at much lower levels than tumor cells (data not shown). Nevertheless, AR suppression in GBM may work both by suppressing tumor cells and by modulating TME, as we have observed in current studies. AR expression levels negatively correlated with the infiltration of CD8 + T cells (*R* = −0.194, p value: 0.0234) but positively correlated with CD4 + T cells (*R* = 0.313, p value: 0.000197) after purity adjustment. These results suggested that higher AR expression levels in tumor may inhibit cytotoxic T cell infiltration (CD8+) but promoting Treg (CD4+). Among the three TGF-βs, only TGF-β2, again, showed consistency with AR, in correlation with both CD8 + and CD4 + T cell infiltration.

Significant positive correlations were seen between TGF-β1 or TGF-β2 with the infiltration of CD4 + T cells (*R* = 0.328, p value: 9.07E-5; and *R* = 0.348, p value: 3.06E-5, respectively). TGF-β2 also negatively correlated with CD8 + T cells infiltration significantly (*R*= −0.205, p value: 0.0164) but the correlations were not significant for TGF-β1. TGF-β1, but not TGF-β2 or TGF-β3, significantly correlated negatively with tumor purity (*R*= −0.361, p-value: 1.37E-5), suggesting that TGF-β1 expressions are more prominent in environmental cells/infiltrated immune cells. TGF-β3 correlates neither with tumor purity nor with T cell infiltration. TGF-β2 appears to be the only member in the family that correlates exclusively with immunosuppressive TME.

### ARA Treatment Reverses the Immunosuppressive Tumor Microenvironment and in Addition, Modulates Systemic Immune Response Affecting T Cell Differentiation in vivo

Immunohistochemistry studies were performed on formalin-fixed and paraffin-embedded (FFPE) mouse brain tumor tissues. RT significantly increased the infiltration of lymphocytes, both CD4 + and CD8 + T cells. ENZA plus RT further increased CD4 + T cells density in the tumor compared with RT only treatment but not so for CD8 + T cells. The most dramatic changes were observed in FoxP3+, the marker for regulatory T cells (Treg) that were significantly decreased in density in the tumor tissue after ENZA treatment alone. ENZA + RT treatment also significantly reduced the otherwise increased FoxP3 + Treg cell density in tumor treated with only RT back to the control level (vehicle treatment only) (Fig. 6B). The density of cells that were positive for CD68, a marker for tumor-associated macrophage (TAM)^39^, was also significantly decreased after ENZA treatment in the tumor tissues. RT alone or ENZA + RT treatment, however, did not affect the density of CD68 + cells in tumor fields (Fig. 6B).

To better understand the mechanism of developing long-term immune memory in mice cured with ARA + RT, we performed additional studies on T cell differentiation in the spleen and peripheral blood in the tumor rechallenge situation (Fig. 6C and Fig. S7). Four weeks after tumor rechallenging/inoculation into the brain of mice that remained tumor-free after the initial ARA + RT treatment, four mice were sacrificed, and tissues were collected for flow cytometry analyses. No significant changes in CD8 + T cell lineage differentiation were observed in peripheral blood (Fig. S7). Although CD4 + T cells increased in percentage overall, the FoxP3 + CD4 + T cells representing Treg and effector memory CD4 + T cells in blood were downregulated, particularly in mice previously treated with BIC (p < 0.05). We also dissected the spleen from mice after rechallenging that were tumor-free after previous treatment with BIC or BIC + RT to study the immunomodulation in the peripheral lymphoid organ (Fig. 6C). Significantly increased proportions of effector memory CD8 + T cells were found in mice previously treated with BIC + RT, but less significantly in those previously treated with BIC only or in control mice without previous treatment. A trend to a significant increase in effector CD8 + T cells is also seen in mice previously treated with BIC + RT. Spleen also was found to sequester more FoxP3 + CD4 + T cells in rechallenged mice previously treated with BIC or BIC + RT, with the same trend seen in FoxP3 + CD8 + T cells. Selected examples of flow cytometry results are summarized in Figure (Fig. S8).

## Discussions

We showed that AR suppression with ARAs, including ENZA or BIC, equally sensitizes brain RT in GBM mice, resulting in 100% long-term survival, compared to 40–50% survival with RT or ARAs alone. To the best of our knowledge, this is the first study on a syngeneic GBM orthotopic mouse model with ARA + RT demonstrating synergistic effects, similar to Werner *et al*., though their model used flank implants in an immunocompromised model^9^. Our results are also consistent with findings of Zalcman *et al* with our advantage of using a syngeneic model instead of nude mice^10,40^. Including ours, there are altogether three animal models that consistently showed radiosensitizing effects of ARAs treating GBM. We also noticed some controversial findings with different tumor responses to orchiectomy between flank and intracranially implanted GBM models by Lathia’s group^41^, which, to us, indeed suggested the advantage of treating GBM with drugs that directly antagonize AR rather than orchiectomy and/or depleting androgen that may induce unknown systemic effects such as negative feedback in the hypothalamuspituitary-adrenal gland axis. Our results are exciting, particularly due to the known resistance of GBM to current standard therapies in clinical practice, including RT and chemotherapy. We have previously studied the role of AR in the cancer stem cells (CSCs) of GBM and demonstrated that AR expression levels are positively correlated with CSC markers and DNA repair enzymes in patients from the TCGA database^12^. We have also demonstrated the preferential suppression of CSCs of ARAs, which is consistent with the current results, confirming that ARAs are radiosensitizers both *in vitro* and *in vivo*^12^.

BIC and ENZA demonstrated very similar outcomes both in vitro and in vivo, suggesting that they interfere with the same target/pathway in GBM. We now further studied the in-depth molecular mechanisms of the radiosensitizing effects of this category of drugs that at least partly explained the findings of dramatically improved tumor control and overall survival/curing of the disease involving immunomodulatory effects from ARA + RT in an orthotopic syngeneic animal model. These findings would not have been revealed if based on studies of combenefit experiments *in vitro* only or by the immunocompromised model(s). Indeed, using 2D/3D cell models along with diverse human GBM cell lines, a mouse-derived cell line, and human primary HGG cultures, we observed a synergistic growth-inhibitory effect ranging from mild to moderate, accompanied by apoptosis-inducing activity. However, *in vivo* experiments using an orthotopic GBM mouse model demonstrated a strong radiosensitizing effect, resulting in prolonged survival/cure.

Interestingly, the combination treatment provided long-term disease control, with all mice surviving over 6 months after RT + ARA without recurrence and prevented tumor growth after re-inoculation of GBM cells. This suggests immune-mediated mechanisms in addition to direct tumor/CSC suppression. This is supported by the immune analysis revealing significantly reduced tumor-associated macrophages (TAM) and regulatory T cells (Treg) in the brain tumor after ARA +/− RT in our syngeneic orthotopic GBM model.

It is exciting to find AR and TGF-β signaling interwined closely, as initially screened out by our RNAseq analysis and now further confirmed with our molecular level of research^12^. The TGF-β signaling pathway plays a key role in cancer progression, metastasis, and resistance to therapy. It acts as a “double-edged sword,” where, in the early stages, it suppresses tumor growth by inhibiting cell proliferation, but in the later stages, it promotes tumor progression by enhancing metastasis and immune evasion^42–44^. Remarkably, TGF-β signaling and Stat3 are intricately connected in GBM, with TGF-β activating Stat3 through both canonical and non-canonical pathways. This connection highlights the potential of targeting either TGF-β or Stat3 axes as a therapeutic strategy in GBM. Mechanistically, Stat3 phosphorylation has been known to play a critical role in modulating several cellular functions and thus contributing to radioresistance in cancer, consistent with our findings^17,18,45,46^. Stat3 phosphorylation has been shown to be induced by TGF-β signaling through Smad-dependent induction of LIF to promote self-renewal capacity of glioma CSCs in patient-derived GBM specimen^16^. The TGF-β/LIF-LIFR/STAT axis has been proposed to be essential in oncogenesis by regulating CSCs in GBM. LIF/LIFR has been demonstrated to play a key role in immunosuppression in the TME, opposing the effects of IL6 on T cells^16,19^. This is particularly important since increased CD4 + and CD8 + T cells were both observed in recurrent GBM compared to the tumor of newly diagnosed, but the TME remains immunosuppressive^47^. Building on this rationale, we investigated the impact of ARAs on the TGF-β/LIF-LIFR/STAT axis and its immune implications in GBM. Our analysis of TGF-β signaling, including both canonical and non-canonical regulation, revealed several promising findings. WB analysis showed that Smad3 phosphorylation, but not Smad2, was consistently altered in mouse and human GBM cells within 8 hours of ARA treatment—preceding Stat3 phosphorylation, which occurred after 24 hours. These findings suggest that ARA-induced Stat3 regulation is likely downstream of TGF-β signaling. AR–TGF-β pathway interactions are established in prostate cancer^48,49^, and our RNA-seq data also identified TGF-β signaling as a top pathway affected by ARA in GBM cells^12^.

**Figure 7:**
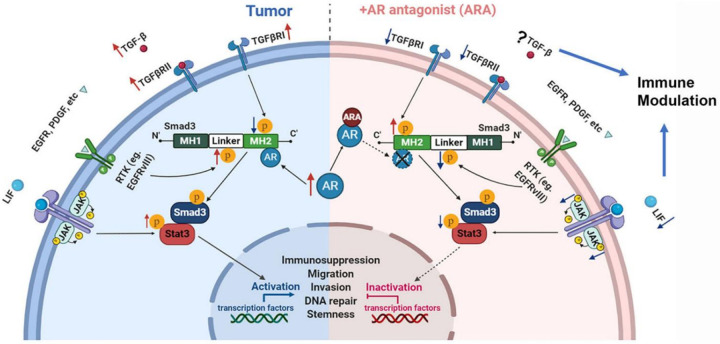
Schematic mechanistic model of AR-Smad3 interaction and impact of ARAs on TGF-β/Smad3 and LIF/STAT3 signaling axes in GBM. Model of AR-Smad3 interaction as a potential mechanism of switching the anti-tumorigenic TGF-β signaling to be pro-tumorigenic by modulating pSmad3L and pSmad3C phosphorylation pattern. Left half: The overexpressed AR in GBM, particularly in CSCs, binds to the C-terminal MH2 domain of Smad3, which decreases pSmad3C from TGF-β signaling, allowing the linker domain phosphorylation of Smad3 (pSmad3L) to dominate its pro-tumorigenic effects. Right half: AR antagonist (ARA) disrupts AR-Smad3 interactions and serves dual functions by promoting pSmad3C and suppressing pSmad3L, thus switching the pro-tumorigenic signaling of the TGF-β pathway in GBM back to tumor suppression as in normal tissue. ↑upregulated; ↓downregulated in the tumor or tumor after ARA.

TGF-β signaling plays a critical role in sustaining CSCs and the immunosuppressive TME, as highlighted by Han *et al*. (2015). TGF-β1 functions as a master multifunctional cytokine in immune regulation, orchestrating pro- and anti-inflammatory responses. In normal epithelial cells, it acts as a tumor suppressor partly through canonical pSmad3C signaling, which inhibits cell growth by counteracting mitogenic signals that promote pSmad3L phosphorylation *via* Ras-associated kinases^50^. During malignant transformation, epithelial or lymphoid cells often become resistant to TGF-β’s growth-inhibitory effects by shifting Smad3 signaling from tumor-suppressive pSmad3C to tumor-promoting pSmad3L. However, the mechanism(s) inducing this shift is largely unknown^51^. Recently Yu *et al* reported that TGF-β1 treatment of U87MG cells suppressed cell growth which could be counteracted by DHT *in vitro*^52^. These findings seemed to contraindicate the results from TGF-β3 suppression studies by Seystahl et al, which could be explained by the differential functions of TGF-β isoforms in different subtypes of GBM, and/or heterogeneity of TGF-β functions in different tumor cells, and/or distinct roles of TGF-βs in tumor stem cells/EMT/invasiveness *vs*. proliferation. Nonetheless, results from Yu *et al* gave a hint that AR signaling may play a role of switching TGF-β signaling, particularly TGF-β1 the dominant ligand isoform in GBM, from anti-proliferation in normal tissues to pro-tumorigenic in cancer. Our data also suggest TGF-β2 might be the most important player associated with AR as an immunosuppressor in tumor environment that is consistent with prior reports^53,54^. Among the TGF-β ligands, we observed increased expression of TGF-β2 in both the ENZA and ENZA + RT groups, in cell models as well as in mouse tissues. This is particularly noteworthy given that a previous study reported that an early generation ARA reversed the suppression of DHT on TGF-β2 expression but not TGF- β1 in prostate cancer^55^, suggesting that AR antagonism may differentially relieve this inhibitory effect depending on the ligand type. Although both TGF-β1 and TGF-β2 are overexpressed in GBM, TGF-β1 is mostly expressed in immune cells and may not be secreted from the tumor cells^53,56^. On the other hand, TGF-β2 is known to be secreted from tumor cells^53^. Together, these observations point toward a potential positive feedback mechanism through which ARAs may modulate the TGF-β/Smad3 signaling axis by restoring its anti-proliferative function, with TGF-β2 emerging as a key autocrine mediator. We further hypothesize that ARAs modulate the TGF-β/Smad3 signaling axis through switching of Smad3 phosphorylation patterns which will be discussed in more details later as illustrated in Fig. 7. It is also interesting to find that TGFBR3, a TGF-β receptor without kinase activities but the most critical receptor potentiating TGF-β2 signaling, is the only receptor out of the three whose expression correlates to AR^57^. How all TGF-β ligands interact with AR in tumor cells *vs* environmental cells, *i.e*., infiltrating immune cells, warrants more detailed studies. Our future directions of research will be focused on TGF-β2 and TGFBR3.

Since TGF-βs are secreted cytokines with context-dependent regulatory functions, we are also considering alternative possible mechanism that ARAs may inhibit TGF-β2 secretion from tumor cells, leading to its intracellular accumulation thus blocking its signaling as a paracrine. This hypothesis supports its potential role as an immunosuppressor^12^. However, mechanistic studies will be required to clarify whether ARAs primarily influence TGF-β2 transcription, secretion dynamics, or both. Further validation in preclinical settings, clinical samples and additional mouse models is needed; however, our observation provides valuable preliminary insight. Our results indicate that AR signaling pathway may provide an alternative, and maybe better, target from TGF-β pathway in treating GBM because of the complex differential functions of each TGF- β ligand.

We have, for the first time to our knowledge, demonstrated that ARA treatment can modulate the phosphorylation status of both the c-terminal and internal linker domain of Smad2 and most significantly of Smad3 with the two phosphorylation sites usually exert opposite effects on cell proliferation/carcinogenesis^50^. Our mechanistic studies, the first to show ARA-induced pSmad3L/pSmad3C switching in human and mouse GBM cells, support the hypothesis that AR overexpression shifts TGF-β signaling from anti- to pro-proliferative, especially in the presence of strong mitogenic signals like EGF/VEGF, as proposed above. Whether other pathways mediate this switch in GBM, and the specific role of AR–Smad3 interaction in CSCs, remains to be explored. It has been demonstrated that AR can directly bind to phosphorylated Smad3 (pSmad3) by Yu *et al* in U87MG cells although no specificity of the type of pSmad3 antibody was provided in the publication. It has been studied decades ago that the MH2 domain of Smad3 (c-terminal) and only this domain binds AR directly in prostate cancer^48^. Smad2 and Smad4 do not interact with AR directly^49^. It also has been reported particularly in hepatocellular carcinoma/liver cirrhosis models that c-terminal phosphorylation of Smad2/3 which is in the MH2 domain, *i.e*., canonical TGF-β signaling, suppresses cell proliferation but the linker domain of Smad2/3 phosphorylation has the opposite function. In our protein–protein interaction analysis using primary human HGG cells, phosphorylated Smad3 (either pSmad3L or pSmad3C or possibly both) was found to interact with AR. However, in the reverse IP using pSmad3C (dual phosphorylation site S423/425,) as the pull-down target, no interaction with AR was detected (data not shown), indicating that AR preferentially interacts with the MH2 domain (c-terminal) of Smad3 which is now bound and blocked by pSmad3C antibody. IP results suggested that c-terminal phosphorylation of Smad3 did not prevent AR-Smad3 interaction but AR-Smad3 binding likely prevented c-terminal phosphorylation of the bound Smad3. Interestingly, ENZA treatment binds to AR and disrupts AR/MH2 domain of pSmad3 interaction, concurrently elevating pSmad3C (S423/425) protein levels. These results suggest that ENZA binding of AR induced structural change of AR and subsequent dissociation of AR Smad3 which may expose the MH2 domain of Smad3, promoting dual phosphorylation at its c-terminus and activation of the pSmad3C–dependent signaling cascade. This shift likely contributes to the pro-apoptotic effect and anti-tumorigenic and effectively redirects the TGF-β/Smad3 signaling pathway from an oncogenic to a tumor-suppressive role as TGF-β signaling normally would do in benign tissues. However, to draw a stronger conclusion, a more in-depth molecular analysis examining each phosphorylation site/combination and its interaction with AR is required.

The distinct downstream effects of different Smad3 phosphorylation combinations remain unclear. We observed time-dependent c-terminal phosphorylation and linker dephosphorylation of Smad3, but not consistently Smad2, after ARA treatment. AR/Smad3 interaction may inhibit MH2 domain phosphorylation and/or promote linker phosphorylation, potentially enhancing tumor cell proliferation and CSC self-renewal. Currently, no available drugs in preclinical or clinical studies can both suppress pSmad3L and enhance pSmad3C as ARAs do in our findings. We propose that ARAs uniquely modulate TGF-β signaling by restoring anti-proliferative c-terminal phosphorylation while inhibiting pro-proliferative linker phosphorylation. ARAs, by targeting the AR “switcher,” may offer more effective tumor control than approaches targeting TGF-β ligands, receptors, or linker phosphorylation alone. The challenges in developing drugs targeting the TGF-β pathway with numerous failed attempts in clinical trials may result from an insufficient understanding of TGF-β signaling and its interaction with AR^58^. Based on our data, suppressing AR with ARAs both inhibited GBM CSCs and switched TME towards an immunostimulating status.

It is encouraging to observe the long-lasting and strong immunological memory particularly in mice treated prior with ARA + RT in the rechallenging study which indicates immunostimulatory effects of ARA. The results, however, were not surprising since most evidence derived from different studies points to a rather immunosuppressive role of androgens in different immune cell types by reducing pro-inflammatory and promoting anti-inflammatory mediators^59^. AR is expressed in majority of neutrophil lineages, CD3+, CD4 + and CD8 + thymocytes with the highest expression in cytotoxic T cells^60–62^. Most interestingly, Treg shows constituent expression of AR after differentiation^63^. DHEA administration can restore normal levels of Tregs in a systemic lupus erythematosus (SLE) mouse model. The transcription factor Foxp3, a key Treg regulator, contains AR binding sites in its DNA locus, potentially explaining Treg expansion following testosterone treatment^63^. In comparison, hypogonadal men have significantly increased proportions of circulating naïve CD4 + T cells which decrease after testosterone replacement therapy^64^. Androgen/AR was also found to promote M2 polarization of alveolar macrophages in lung although whether the effects are direct or indirect is unclear^65^. ARA may indirectly suppress LIF expression/secretion in tumor cells by reversing the immunosuppressive TME. While overall TGF-β protein levels in GBM cells may remain unchanged or increased, extracellular matrix levels could be affected, reflecting responses in surrounding cells. It is possible that suppressions of secreted LIF and/or TGF-β are necessary for altering the TME, as suggested in the proposed model (Fig. 7). Fascinatingly, the increased accumulation of effector memory T cells in the spleen of rechallenged mice after BIC + RT suggests that prior ARA treatment contributes to strong, long-lasting immunological memory. This observation aligns with the response of Stat5b to ARA, as shown in Fig. S4. Stat5b, the dominant expressive form of Stat5 downregulated by ARA, is known to promote the development and function of CD4 + FoxP3 + regulatory T cells. Our results are consistent with the previous reports showing dramatically decreased FoxP3 + immune cells in ARA-treated tumor tissues, offsetting the promoting effects of Treg by RT. The combined immunological effects of ARA + RT are only observable in vivo, which helps us understand why a strong synergy was observed in the syngeneic mouse model, but only limited effects were observed *in vitro*. Nonetheless, it remains unclear whether the immune modulatory effects we observed in our studies are simply effects of ARA in TME only or in combination with systemic effects, the detailed study of which is beyond the scope of current project.

### Limitations of the study

Our study does have limitations. In our systemic response studies after ARA + RT, we only focused on T cell lineage but not yet studied other immune cells such as monocytes/macrophages. Neither did we look at the thymus, which is the primary site of T cell differentiation, whose size can be influenced by AR^64^. Although the TIMER studies suggested AR expression levels are more prominent in tumor cells than environmental cells such as the infiltrated immune cells while TGF-β1 is the opposite. The relationship between tumor cells/CSCs and immune cells in the TME in response to ARA ± RT remains unexplored and warrants future investigation. It is unclear whether LIF downregulation alone accounts for the observed TME changes, and whether ARA affects LIFR expression in immune cells. Additionally, our current studies used whole brain RT, while partial brain RT with image-guided treatment and the Small Animal Radiotherapy Research Platform (SARRP) could provide further insights into distal intracranial immune responses. We also acknowledge that U87MG cells have limitation by their long-term culture history and the uncertainty surrounding their origin originally derived in Sweden, the commonly used U87MG cells distributed by ATCC were adapted in the U.S. and may differ significantly from the primary tumor they were meant to model. Although we included primary human HGG cultures to provide proof of the mechanistic concept. However, given the rich database derived from U87MG cell line, and the consistency we observed with other human cell lines as published before^12^, it provides valuable information for guiding future studies in primary human GBM cultures and clinical specimen.

## Conclusion

AR antagonists enhance the efficacy of brain radiotherapy in syngeneic mice with orthotopic GBM, showing strong *in vivo* synergy despite limited *in vitro* effects. This synergy extends beyond direct tumor cell killing, as ARA or ARA + RT treatment induced durable immunological memory. ARA promoted immunostimulation both locally in the TME and systemically, with increased effector memory T cells in the spleen months post-treatment. Mechanistically, ARA acts through modulation of the TGF-β/Smad3 and LIF/STAT3 pathway, impacting both CSC proliferation and immune regulation. Collectively, our results support the combination of ARAs with radiotherapy as a promising and clinically translatable treatment approach for GBM.

## Supplementary Files

This is a list of supplementary files associated with this preprint. Click to download.

- RepBlotsCDD01112026sgnkm.docx
- SupplementaryInformationCDD01112026.docx
- floatimage1.png

## Data Availability

All data supporting the findings of this study are included in the main manuscript and Supplementary Materials. Additional information related to this manuscript is available from the lead author contact upon request.

## Highlights

AR antagonists (ARAs) have radio-sensitizing effects on GBM.

AR antagonists synergize radiotherapy with combined treatment achieving 100% overall survival in an orthotopic GBM mouse model.

ARA disrupts AR binding to pSmad3, leaving the MH2 domain accessible and facilitating the c-terminal dual phosphorylation of Smad3 (pSmad3C (S423/425)) by TGF-β signaling.

ARA plus radiotherapy promotes immunostimulation in the tumor microenvironment and systemically.

ARA plus radiotherapy may serve as a novel treatment for GBM in the clinic.

## Abbreviations

GBM: Glioblastoma
AR: Androgen Receptor
ARAs: Androgen receptor antagonists
RT: Radiotherapy
BIC: Bicalutamide
ENZA: Enzalutamide
TME: Tumor Microenvironment
CSCs: Cancer Stem Cells
TGF-β: Transforming Growth Factor-beta (TGF-β)
GSEA: Gene Set Enrichment Analyses
TCGA: The Cancer Genome Atlas
UPLC: Ultra-Performance Liquid Chromatography
MRI: Magnetic Resonance Imaging
WB: Western Blotting
IP: Immunoprecipitation
IHC: Immunohistochemistry
FACS: Fluorescence-Activated Cell Sorting
HGG: High Grade Glioma
EMT: Epithelial-Mesenchymal Transition
TAM: Tumor-Associated Macrophages
Treg: regulatory T cells
SLE: Systemic Lupus Erythematosus
SARRP: Small Animal Radiotherapy Research Platform
IVIS: In Vivo Imaging System
FFPE: Formalin-Fixed Paraffin-Embedded
